# Supplementary material for: A Brain to Spine Interface for Transferring Artificial Sensory Information
Source: Sci Rep. 2020 Jan 21;10:900. doi: 10.1038/s41598-020-57617-3 (PMC6972753; doi:10.1038/s41598-020-57617-3)
Supplement: Supplementary file 1 — Supplementary Information. [file 41598_2020_57617_MOESM1_ESM.docx]

# Supplementary Information

# A Brain to Spine Interface for Transferring Artificial Sensory Information

Amol P. Yadav^1,2,3^, Daniel Li^2,3^, Miguel A. L. Nicolelis*^1-8^

Department of Neurosurgery^1^, Department of Neurobiology2, Department of Biomedical Engineering3, Department of Psychology and Neuroscience4, Department of Neurosurgery5, Department of Neurology6, Duke Center for Neuroengineering7, Duke University, Durham, NC, 27710; Edmund and Lily Safra International Institute of Neuroscience8, Natal, Brazil 59066060.


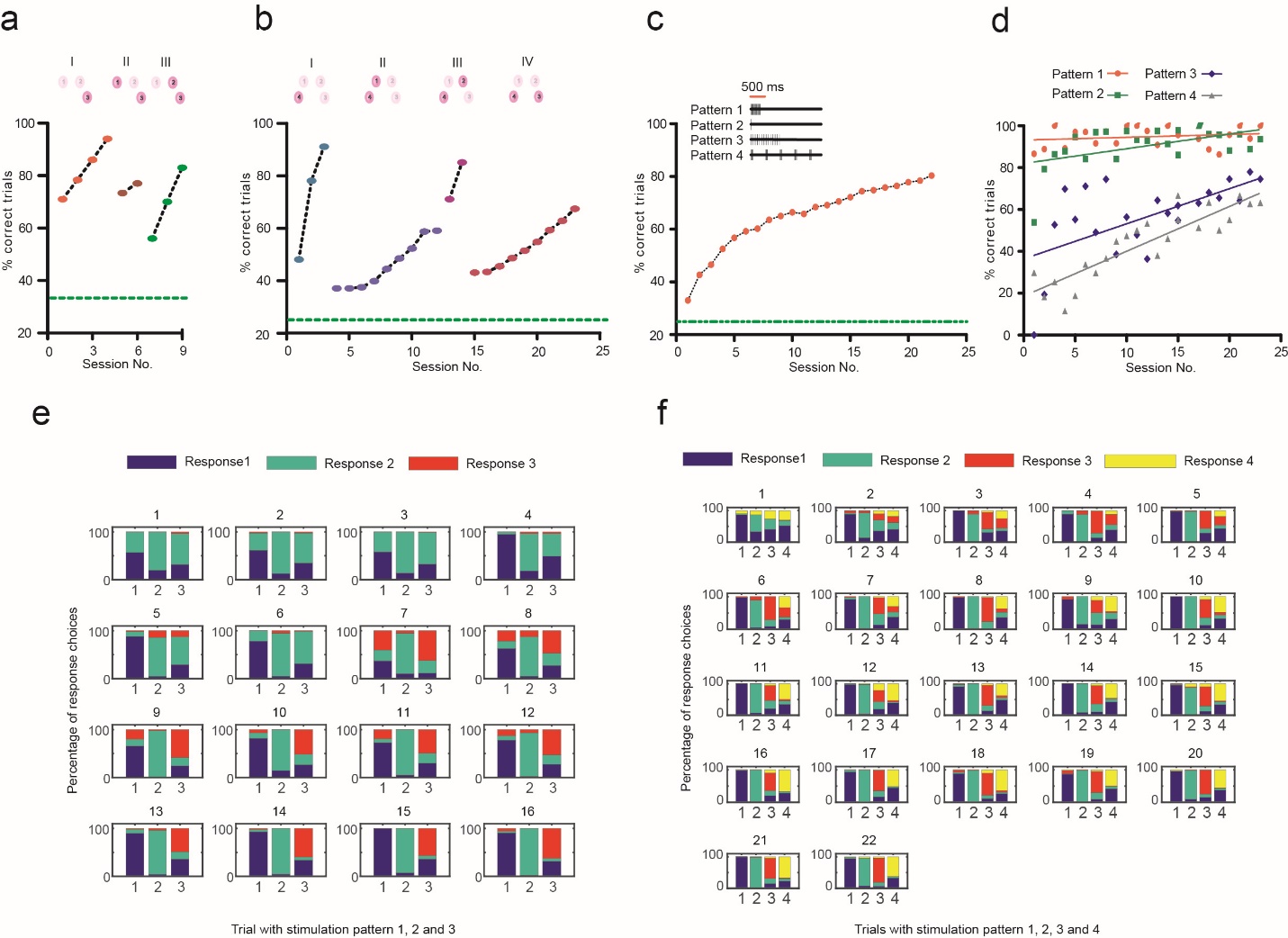


**Supplementary Figure 1. a)** Training paradigm to associate novel stimulation pattern (Pattern 3) with reward port 3 for one rat. In stage I, all trials in the session belonged to Pattern 3 (100 pulses at 100Hz). During stages II and III, trials for both Pattern 3 and Pattern 1, and Pattern 3 and Pattern 2 were respectively applied in the training sessions. Once the rat performed >75% correct in stages II and III, training sessions with all three patterns together were initiated as shown in Fig 2c. Chance performance was kept at 33% during all stages by giving the rat access to make a nose poke at any of the three reward ports (green line). **b)** Training paradigm to associate novel stimulation pattern (Pattern 4) with reward port 4 for one rat. In stage I, all trials in the session belonged to Pattern 4 (100 pulses in 5 bursts of 20 pulses each with inter-burst frequency of 2 Hz and inter pulse frequency of 333 Hz). During stages II, III, and IV, trials for both Pattern 4 and Pattern 1, Pattern 4 and Pattern 2, and Pattern 4 and Pattern 3, were respectively applied in the training sessions. Once the rat performed >60% correct in stages II, III, and IV, training sessions with all four patterns together were initiated. Chance performance was kept at 25% during all stages by giving the rat access to make a nose poke at any of the four reward ports (green line). **c)** Learning curve showing one rat learned to discriminate 4 DCS patterns (Pattern 1: 100 pulses at 333 Hz, Pattern 2: 1 pulse, Pattern 3: 100 pulses at 100 Hz, and Pattern 4: 100 pulses in five bursts of 20 pulses each). Graph shows percentage correct trials for 22 sessions, green line represents chance level of 25 %). **d)** Discrimination performance for each of the four patterns shown individually across sessions. **e)** Percentage of response choices classified by trial type for one rat as it learned to discriminate three stimulation patterns. Stacked bar graphs are color coded for response type. Trial type is indicated on x-axis. Notice how % response 1 on trial type 1, % response 2 on trial type 2, and % response 3 on trial type 3, increases from session 1 to 16. **f)** Percentage of response choices classified by trial type for one rat as it learned to discriminate four stimulation patterns. Stacked bar graphs are color coded for response type. Trial type is indicated on x-axis. Notice how % response 1 on trial type 1, % response 2 on trial type 2, % response 3 on trial type 3, and % response on trial type 4, increases from session 1 to 22.
